# Comparative *in-silico* proteomic analysis discerns potential granuloma proteins of *Yersinia pseudotuberculosis*

**DOI:** 10.1038/s41598-020-59924-1

**Published:** 2020-02-20

**Authors:** Manisha Aswal, Anjali Garg, Neelja Singhal, Manish Kumar

**Affiliations:** Department of Biophysics, University of Delhi South Campus, New Delhi, 110021 India

**Keywords:** Functional clustering, Genome informatics

## Abstract

*Yersinia pseudotuberculosis* is one of the three pathogenic species of the genus *Yersinia*. Most studies regarding pathogenesis of *Y. pseudotuberculosis* are based on the proteins related to Type III secretion system, which is a well-known primary virulence factor in pathogenic Gram-negative bacteria, including *Y. pseudotuberculosis*. Information related to the factors involved in *Y. pseudotuberculosis* granuloma formation is scarce. In the present study we have used a computational approach to identify proteins that might be potentially involved in formation of *Y. pseudotuberculosis* granuloma. A comparative proteome analysis and conserved orthologous protein identification was performed between two different genera of bacteria - *Mycobacterium* and *Yersinia*, their only common pathogenic trait being ability to form necrotizing granuloma. Comprehensive analysis of orthologous proteins was performed in proteomes of seven bacterial species. This included *M. tuberculosis*, *M. bovis* and *M. avium paratuberculosis* - the known granuloma forming *Mycobacterium* species, *Y. pestis* and *Y. frederiksenii* - the non-granuloma forming *Yersinia* species and, *Y. enterocolitica* - that forms micro-granuloma and, *Y. pseudotuberculosis* - a prominent granuloma forming *Yersinia* species. *In silico* proteome analysis indicated that seven proteins (UniProt id A0A0U1QT64, A0A0U1QTE0, A0A0U1QWK3, A0A0U1R1R0, A0A0U1R1Z2, A0A0U1R2S7, A7FMD4) might play some role in *Y. pseudotuberculosis* granuloma. Validation of the probable involvement of the seven proposed *Y. pseudotuberculosis* granuloma proteins was done using transcriptome data analysis and, by mapping on a composite protein-protein interaction map of experimentally proved *M. tuberculosis* granuloma proteins (RD1 locus proteins, ESAT-6 secretion system proteins and intra-macrophage secreted proteins). Though, additional experiments involving knocking out of each of these seven proteins are required to confirm their role in *Y. pseudotuberculosis* granuloma our study can serve as a basis for further studies on *Y. pseudotuberculosis* granuloma.

## Introduction

The genus *Yersinia* is comprised of Gram-negative, catalase-positive, facultative anaerobic enteric-bacteria. Though, the optimal temperature for growth is 28 °C, some members of the genus can survive at low temperatures *ca*. 4 °C^1^. Most species of the genus *Yersinia* grow extracellularly, except *Y. pseudotuberculosis* and *Y. pestis* which are capable of intracellular growth, *i.e*. inside the host macrophages^2^. Of the sixteen known species of *Yersinia*, only three are pathogenic *Y. pestis*, *Y. pseudotuberculosis* and *Y. enterocolitica*^3,4^. In humans, infection with *Y. pestis* results in plague, while infection with *Y. pseudotuberculosis* and *Y. enterocolitica* results in gastroenteritis, which is usually self-limiting^5^. Besides man, *Y. pseudotuberculosis* can infect a wide range of animals including swines, dogs, rodents, birds *etc*.^6–9^. *Y. pseudotuberculosis* infection in animals can lead to tuberculosis-like symptoms, including localized tissue necrosis and granulomas in the liver, spleen, and lymph nodes.

Plasmid-encoded *Yersinia* outer proteins (Yops) have been regarded as an essential virulence factor of the pathogenic *Yersinia* spp., which restrain the host immune mechanisms to the local lymph nodes. When *Yersinia* and the target host cell come in mutual contact, Yops are delivered in the host cells with the help of type III secretion system (T3SS)^10^. Since polymorphonuclear neutrophils are the first cells to reach the infection site, these are regarded as the main targets of Yps T3SS-mediated Yops translocation^11^. Though, multiple factors underlie the virulence mechanism, the current knowledge about virulence of *Y. pseudotuberculosis* is based mainly on the secretion systems. To date, information about *Y. pseudotuberculosis* proteins, which help in granuloma formation, sustenance, expansion and dissemination in the host is fragmentary. Thus, the present study was conducted to identify *Y. pseudotuberculosis* proteins involved in granuloma formation using a system biology approach.

The present study is based on three *Mycobacterium* spp. that included *M. tuberculosis* (Mtb), *M. bovis* (Mbov) and *M. avium paratuberculosis* (Map) - the known granuloma forming *Mycobacterium* species, *Y. pestis* (Ype) and *Y. frederiksenii* (Yfr) - the non-granuloma forming *Yersinia* species and, *Y. enterocolitica* (Yen) - that forms micro-granuloma and, *Y. pseudotuberculosis* (Yps) - a prominent granuloma forming *Yersinia* species. We have considered Yen as non-granuloma forming since it forms non-prominent micro-granuloma, unlike Yps, which forms a prominent granuloma. Despite, the fact that Mtb and Yps belong to two different microbial genera, the granuloma formed by both share several common features: (i) both form a similar type of granuloma in the host which is both necrotizing and infectious, different from other forms of granuloma, (ii) the pathological symptoms of *Yersinia* infection *i.e*. granulomatous ileitis, colitis and appendicitis (causative agent - Yen and Yps) are similar with the symptoms of tuberculosis (causative agent – Mtb) (iii) cellular infiltration of neutrophils and histiocytes is found in the lesions produced by both Yps and mycobacteria^12–15^. Despite many similarities, there are also a few differences between tubercular and pesudotubercular granuloma *viz*. (i) mycobacterial lesion (non-suppurative) involves activation of T-cell mediated immune response^16,17^ while in a *Yersinia* lesion (suppurative) despite the presence of T-cells and macrophages, there is suppression of T- and B-cells (adaptive immunity) due to the release of virulence factors^18,19^ (ii) both form a central necrotic granuloma, the structure underlying necrotic tissue is maintained in *Yersinia* granuloma but is inconspicuous in a tuberculous granuloma^17^. Along with Yps, Yen also causes granulomatous ileitis, colitis and appendicitis. Researchers have reported that evident granuloma are formed in case of *Mycobacterium* species, Yps, *Chlamydia* species and *Treponema* species, whereas micro-granulomas are formed by Yen, *Salmonella* spp. and *Campylobacter* spp. Yps infection is characterized by a granulomatous process with central micro abscess, while Yen granulomas are accompanied by an acute inflammation and hemorrhagic necrosis^20^. Also, it has been suggested that Yen infection in gastrointestinal tract is usually not associated with granuloma. Infection with Yen has been characterized by mucosal ulceration, often initially overlying Peyer’s patches, with accompanying hemorrhagic necrosis, palisading histiocytes and lymphoid hyperplasia^21–23^. Gastrointestinal infection with Yps is usually described as a granulomatous process with central micro abscesses, and almost always accompanied by mesenteric adenopathy^23–25^.

In the present study, similarities and differences were discerned in the proteomes of seven bacteria using a bottom-up approach. The proteomes that were compared, included proteomes of three mycobacterial spp. (*M. tuberculosis* H37Rv*, M. bovis* ATCC BAA-935/AF2122/97 and *M. avium paratuberculosis* ATCC BAA-968/K-10 and four *Yersinia* spp. like *Y. pestis* CO-92/Biovar *orientalis, Y. enterocolitica* NCTC 13174/8081, *Y. pseudotuberculosis* IP 31758 and *Y. frederiksenii* ATCC 33641. The primary step was to define the core proteome, which was done using sequence homology. Gradually, we narrowed down our study towards species-specific proteomes. The rationale of our study was based on following basic premises: (a) proteins, which are present in all the seven proteomes, should be the part of conserved core gene set and should be involved in house-keeping functions, (b) proteins which are present in all the species of either *Yersinia* or mycobacteria should also be involved in genus-specific house-keeping functions and, (c) if we remove the proteins of category (a) and (b) the only common proteins between Yps and *Mycobacterium* spp. might be the proteins which help in granuloma formation, as these proteins are not shared with other species of *Yersinia*. In the present work, we have proposed the proteins of category (c) as putative granuloma forming proteins. Functional annotation of these proteins suggested involvement of these proteins in granuloma formation in Yps.

## Results

### Comparative analysis of genome and proteome relatedness

**Figure 1:**
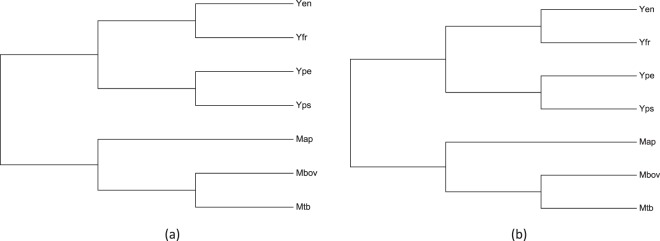
Relatedness among the seven bacterial species on the basis of their genome and proteomes. Cladograms were generated using Neighbor-Joining method^78^ using (**a**) average nucleotide identity (ANI) and (**b**) Percentage of conserved proteins (PCOP).

On the basis of average nucleotide indentity (ANI) of genomes and percentage of conserved proteins (PCOP) content of proteomes, relatedness among all the seven bacterial species was analyzed. Figure 1(a,b) shows relationships among the seven genomes and proteomes, respectively. Both the genomic and proteomic trees divided all the species into two branches; one specific to *Yersinia* spp. and the other to *Mycobacterium* spp. Among the four *Yersinia* spp. Yen and Yfr formed a common cluster while Ype and Yps formed a separate cluster. Among *Mycobacterium* spp., Mbov and Mtb were present in one cluster, and Map was present in a separate cluster.

### Comparative analysis of proteomes of mycobacteria and *Yersinia*

A pairwise comparative analysis between different proteomes revealed that Mbov (causative agent of bovine TB) and Mtb (causative agent of human TB) shared 3845 proteins. Map shared 2602 and 2632 proteins with Mbov and Mtb, respectively (Table 1). Interestingly, Map shared more protein with Yen (926) and Yfr (959), than with Yps (896) and Ype (882). Among the different species of *Yersinia*, the two top most closely related proteomes on the basis of number of shared proteins, were Yps and Ype (number of shared proteins was 3387), and, Yen and Yfr (number of shared proteins was 3304).

**Table 1:** Total proteome size of the seven microbes and the number of shared orthologous proteins.

S. No.	Microbe	Number of proteins in complete proteome	Number of shared orthologous proteins						
			Mbov	Mtb	Map	Yen	Yfr	Ype	Yps
1	Mbov	3976	—	3845	2602	892	917	868	868
2	Mtb	3993		—	2632	893	919	872	874
3	Map	4316			—	926	959	882	896
4	Yen	4026				—	3303	2809	2885
5	Yfr	3909					—	2789	2933
6	Ype	4354						—	3387
7	Yps	4305							—

Mbov- *M. bovis*, Mtb- *M. tuberculosis*, Map- *M. avium paratuberculosis*, Yen- *Y. enterocolitica*, Yfr- *Y. frederiksenii*, Ype- *Y. pestis*, Yps- *Y. pseudotuberculosis*.

### Clustering of orthologous proteins

**Figure 2:**
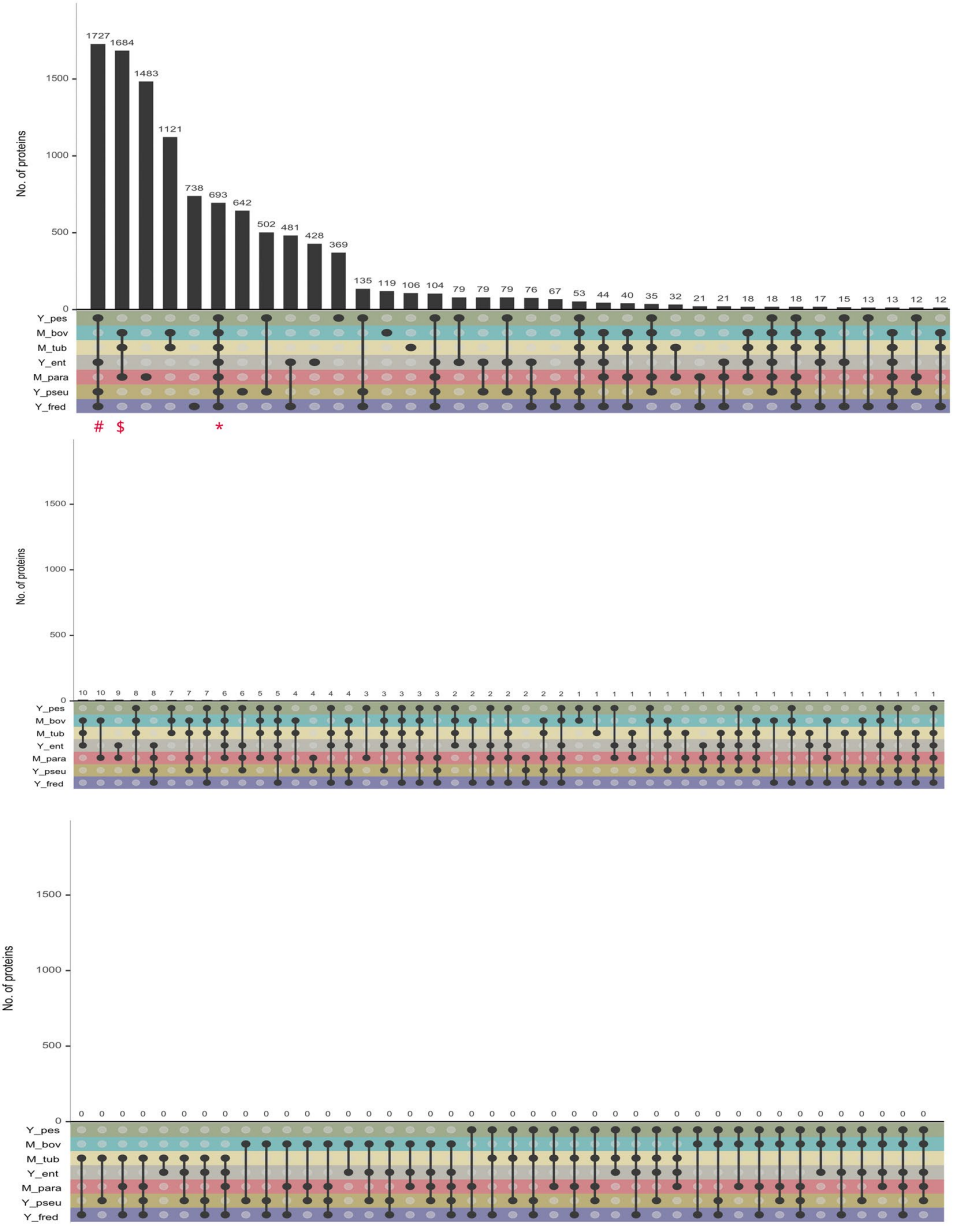
Number of proteins in mutually exclusive protein clusters formed due to different combinations of seven proteomes (*M. tuberculosis, M. bovis, M. paratuberculosis* and non-granuloma forming *Yersinia* species like *Y. pseudotuberculosis, Y. enterocolitica and Y. frederiksenii*). Solid dots show presence of proteins in the corresponding proteome and line between two solid dots shows presence of orthologous proteins in the two proteomes. The vertical bars show the number of shared homologous proteins (orthologous proteins) among the proteomes. Single solid dot represents species unique proteins. The plot was drawn from UpSet plot tool **(**https://gehlenborglab.shinyapps.io/upsetr/**)**. Panel (a–c) Shows combination of proteomes which shared >10, ≤10 and 0 orthologous proteins respectively^85^. ‘*’ represent proteins shared among all seven proteomes, ‘$’ and ‘#’ shows intra-genus conserved proteins of *Mycobacterium* spp. and *Yersinia* spp respectively.

All the possible combinations of the proteomes yielded 127 different protein clusters, of which only 85 clusters contained proteins (Fig. 2). An inter-genus comparison of proteins conserved across the seven proteomes resulted in a conserved set of 693 proteins. We also performed an intra-genus conservation analysis and found 1684 proteins for *Mycobacterium* spp. and 1727 proteins for *Yersinia* spp. The number of proteins which were present only in Yps, Ype, Yfr, Yen, Mtb, Map and Mbov were 642, 369, 738, 428, 106, 1483 and 119, respectively.

### Functional analysis of orthologous protein cluster

**Figure 3:**
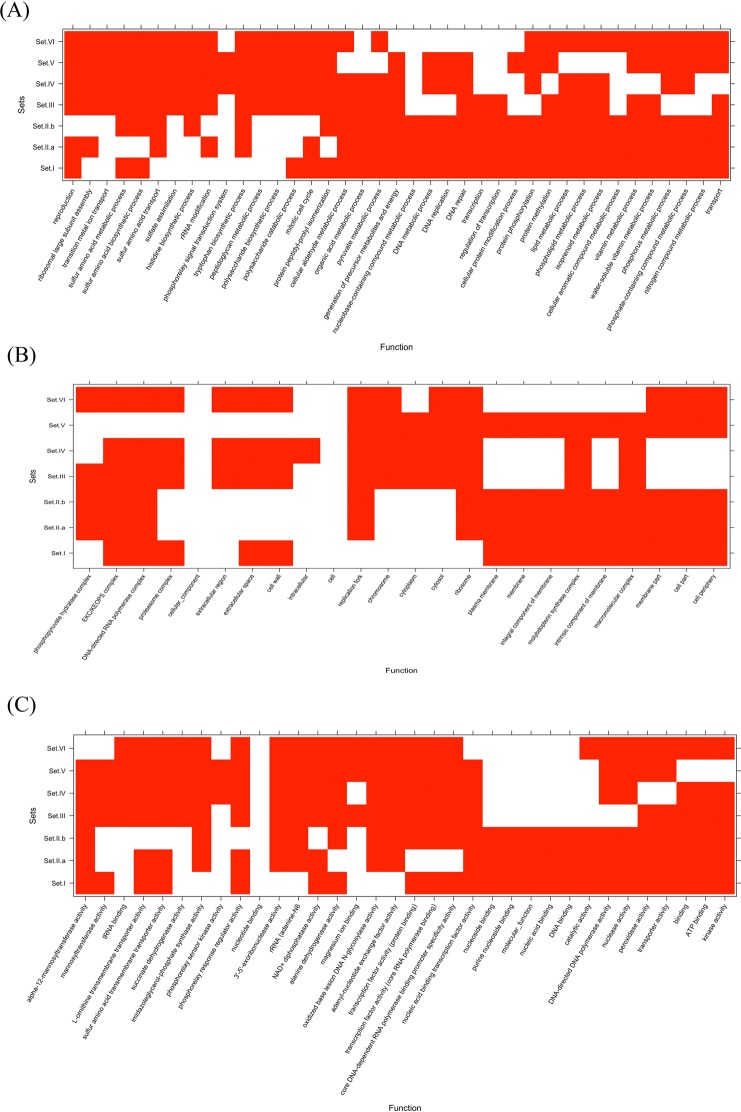
Functional enrichment analysis of different mutually exclusive protein clusters. The enrichment analysis was done for six sets, Set I - inter-genus, Set II - intra-genus: (**A**) *Mycobacterium* spp. and (**B**) *Yersinia* spp., and *Mycobacterium* spp. with Ype Set III; Yen Set IV; Yps Set V and Yfr Set VI. Top 10 enriched GO terms of GO Biological Processes (BP), GO Cellular Components (CC), and GO Molecular Functions (MF) are shown in panels a–c, respectively. Red color shows absence of function where as white shows presence.

Inter genus analysis of proteomes revealed presence of a conserved set of 693 proteins in the seven proteomes. Presence of these 693 proteins across the seven proteomes indicated their involvement in vital functions of the seven microbes. This also implied that these 693 proteins might be considered as the core proteome. To validate this assumption, we analyzed the top 10 enriched GO terms of biological processes associated with these proteins. Enrichment analysis revealed involvement of these 693 proteins in carbohydrate biosynthesis, amino acid biosynthesis and transport, sulfur metabolism, cell wall biosynthesis and overall metabolic processes (Fig. 3). Thus the GO term enrichment analysis also validated our assumption that these proteins were involved in housekeeping functions. Among the 1684 proteins that were conserved only in *Mycobacterium* spp., the enriched GO terms of biological process was biosynthesis of sulfur containing amino acids (cysteine and methionine) and metabolic proteins, in addition to the functions that were enriched in inter-genus core protein set. The 1727 proteins conserved only in *Yersinia* spp., were mainly metabolic and reproduction/mitotic cell cycle proteins. Yen shared 18 proteins with *Mycobacterium* spp. (Mbov, Map and Mtb). These proteins were mostly related to vitamin metabolism, DNA regulation and transport. While, Yfr and *Mycobacterium* spp. (Mbov, Map and Mtb) shared 40 proteins, which were mainly involved in DNA replication, repair, regulation, and metabolism. The five proteins, which were common between Ype and *Mycobacterium* spp. (Mbov, Map and Mtb) were involved in metabolism, DNA replication and protein modification. Interestingly, it was observed that seven proteins were common between Yps and *Mycobacterium* spp. (Mbov, Map and Mtb). These proteins were involved in lipid metabolism, cell wall synthesis and, pyruvate and aldehyde metabolism. It is pertinent to mention here that proteins of each ortholog cluster were mutually exclusive. For example, the seven proteins common among Yps and *Mycobacterium* spp. were not a part of any other ortholog clusters.

### Functional characterization of common orthologs of Yps and *Mycobacterium spp*. and their probable involvement in granuloma formation

A comparative analysis of protein conservation in the three *Mycobacterium* spp. and four *Yersinia* spp. revealed that seven Yps proteins were present in the three *Mycobacterium* spp. but absent in other species of *Yersinia*. Since, the only common feature in the three *Mycobacterium* spp. and Yps is their capability to form macro-granuloma, it might be anticipated that these seven proteins might play a potential role in granuloma formation (Table 2). To validate the role of these proteins in Yps granuloma, a detailed functional characterization of all the seven proteins was performed using UniProtKB, STRING and KEGG databases. It was observed that, of the seven proteins, two proteins were functionally uncharacterized, while functions of five proteins were known. The details of the seven proteins with their UniProt ids, interaction partners and pathway information analysis are presented in Table 3.

**Table 2:** Characterization of protein domains present in Yps granuloma proteins, interacting proteins and metabolic pathways as discerned using UniProtKB, STRING and KEGG, respectively.

S. No.	UniProt ID	Protein Name	Domain		STRING Annotation			KEGG pathway
			Position(s)	Description	Definition	Protein	E-value	
1.	A0A0U1QT64	Uncharacterized protein	9–134	AAA - ATPases associated with a variety of cellular activities	—	—	—	—
2.	A0A0U1QTE0	Uncharacterized protein	64–307	Formylglycine-generating sulfatase enzyme	—	—	—	—
3.	A0A0U1QWK3	ABC transporter, ATP-binding protein	8–239, 332–561	ABC transporter	ABC transporter family protein	yadG	2.8e-33	—
4.	A0A0U1R1R0	5-carboxymethyl-2-hydroxymuconate semialdehyde dehydrogenase (EC 1.2.1.60)	16–474	Aldedh- Aldehyde dehydrogenase family	5-carboxymethyl-2-hydroxymuconate semialdehyde dehydrogenase	hpaE	5.7e-288	Tyrosine metabolism Microbial metabolism in diverse environments Degradation of aromatic compounds
5.	A0A0U1R1Z2	Uncharacterized protein	30–295	Cellulase - glycoside hydrolase family 5	glycosyl hydrolase 10 family protein	DJ40_3168	6.6e-225	—
6.	A0A0U1R2S7	Transcriptional regulator, TetR family	14–74	tetR- DNA-binding, helix-turn-helix (HTH) domain	bacterial regulatory s, tetR family protein	yxaF	6.6e-115	—
7.	A7FMD4	4-hydroxy-3-methylbut-2-enyl diphosphate reductase	—	—	4-hydroxy-3-methylbut-2-enyl diphosphate reductase	ispH	5.2e-179	Terpenoid backbone biosynthesis Metabolic pathways Biosynthesis of secondary metabolites Biosynthesis of antibiotics

**Table 3:** Information about the interacting proteins of the potential Yps granuloma proteins, the various metabolic pathways in which they are involved and, the proposed drug targets.

S. No.	UniProt ID	Interacting proteins	KEGG pathway analysis of the interacting proteins	Interacting proteins/pathways proposed as potential drug-targets
1.	A0A0U1QT64	Uncharacterized protein	—	—
2.	A0A0U1QTE0	Uncharacterized protein	—	—
3.	A0A0U1QWK3	yadH queF can icaB lepB gstB ybhS	Folate biosynthesis, Metabolic pathways (queF), Nitrogen metabolism (can), Protein export (lepB), Glutathione metabolism (gstB)	Glutathione metabolism^40,86^, ABC transporters^87^
4.	A0A0U1R1R0	hpcD hpaD hpcE_1 hpcE_2 hpaH hpaI hpaR hpaX hpaB nifJ	Tyrosine metabolism, Microbial metabolism in diverse environments, Degradation of aromatic compounds (hpcD, hpaD, hpcE_1, hpcE_2, hpaH, hpaI, hpaB) Glycolysis/Gluconeogenesis, Citrate cycle (TCA cycle), Pyruvate metabolism, Butanoate metabolism, Metabolic pathways, Biosynthesis of secondary metabolites, Microbial metabolism in diverse environments, Biosynthesis of antibiotics, Carbon metabolism (nifJ)	D-Alanine metabolism^88^, Metabolic pathways^89^, tyrosine metabolism, pyruvate metabolism, Butanoate metabolism, Glycolysis/Gluconeogenesis, Citrate cycle (TCA cycle)^87^
5.	A0A0U1R1Z2	nhaR melB2	_	_
6.	A0A0U1R2S7	DJ40_975 rpsC tatD tesB purC tmk icIR	Ribosome (rpsC), Biosynthesis of unsaturated fatty acids (tesB), Purine metabolism, Metabolic pathways, Biosynthesis of secondary metabolites, Biosynthesis of antibiotics (purC) Pyrimidine metabolism, Metabolic pathways (tmk)	Fatty acid biosynthesis, Purine metabolism, pyrimidine metabolism^87^
7.	A7FMD4	ispG ispA lspA rpsA cmk ispF ispD dxs fkpB dxr	Terpenoid backbone biosynthesis, Metabolic pathways, Biosynthesis of secondary metabolites, Biosynthesis of antibiotics (ispG, ispF, ispD, dxs, dxr), Protein export (lspA), Ribosome (rpsA), Pyrimidine metabolism, Metabolic pathways (cmk), Thiamine metabolism (dxs),	Thiamine metabolism^90^ Terpenoid backbone biosynthesis^63,64^

### Validation of the identified putative granuloma proteins with the gene expression data

To validate the expression status of the seven Yps granuloma proteins, RNAseq gene expression dataset (ID GSE55292) of *Yersinia pseudotuberculosis* YPIII strain NC_010465.1^26^ was obtained from the GEO database^27^. We observed that, of the seven proteins, five proteins were expressed *in-vitro* (above 90% sequence identity). However, the expression of remaining two proteins (UniProt id: A0A0U1QT64 and A0A0U1QTE0) could not be ascertained.

### Validation of the role of identified Yps granuloma proteins using experimentally identified Mtb granuloma proteins

**Figure 4:**
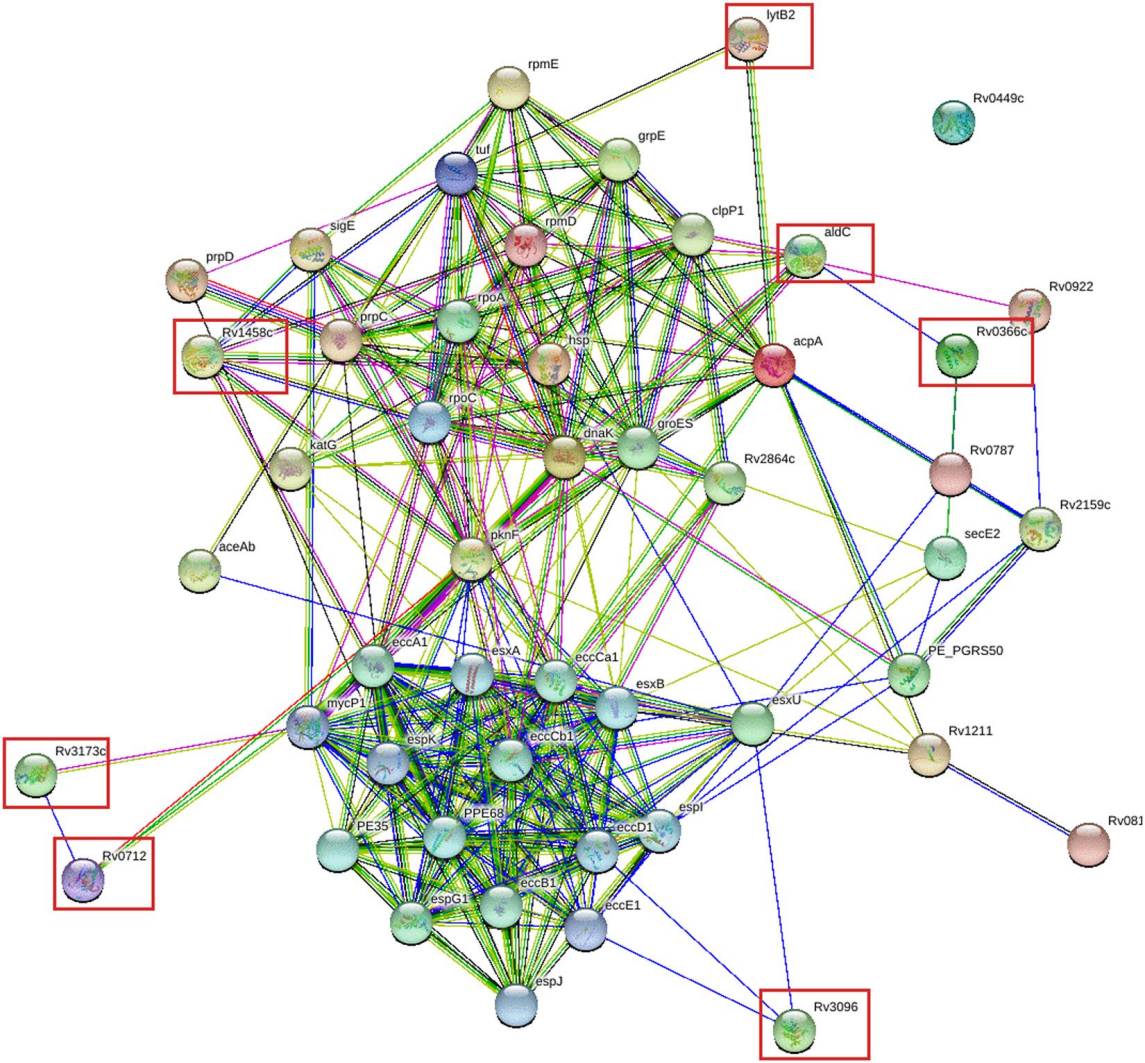
A composite protein-protein interaction network map of Mtb RD1 locus proteins, ESAT-6 secretion system proteins and intra-macrophage secreted proteins, constructed using STRING database.The Mtb orthologs of the seven proposed Yps proteins (Rv0366c, Rv0712, Rv1458c, Rv2858c (aldc), Rv3096, Rv3173c, and Rv3382c (lytB2)) are marked in red colored rectangular boxes. The colour and thickness of edges (lines connecting two proteins or nodes) indicates type and confidence of interaction, respectively. The color coding of edges are as follows: Red line - indicates the presence of fusion evidence; Green line - neighborhood evidence; Blue line - cooccurrence evidence; Purple line - experimental evidence; Yellow line - textmining evidence; Light blue line - database evidence; Black line - coexpression evidence.

Earlier studies have proved that Mtb RD1 locus proteins^28^, ESAT-6 secretion system proteins^29^ and intra-macrophage secreted proteins^30^ play an important role in the formation and regulation of granuloma. To ascertain the probable involvement of the seven proposed Yps proteins in Yps granuloma formation, we constructed a composite protein-protein interaction (PPI) network map of the Mtb RD1 locus proteins, ESAT-6 secretion system proteins and intra-macrophage secreted proteins. The orthologs of the proposed Yps granuloma proteins in the Mtb proteome were mapped on the composite PPI network. Interestingly, all the mapped proteins showed moderate to strong connections with other proteins of the composite PPI network (Fig. 4).

## Discussion

Identification of the orthologous protein(s)/gene(s) is a useful method for determining relatedness among different taxonomic groups *viz*. genera, species and strains. In the present study this approach was used to identify Yps proteins, which might be involved in granuloma formation. A comparative *in-silico* analysis of the conserved orthologs of Yen, Ype, Yps, Yfr, Map, Mbov and Mtb proteomes was performed to predict proteins that might be involved in Yps granuloma formation. Initially, we analyzed the genomic and proteomic relatedness among the seven species. At genomic level, analysis was done using pair-wise comparison of ANI values, which is routinely used as a measure of overall similarity between two genome sequences^31^. Results of the present study reiterated the results of previous phylogenetic studies, that the number of shared homologs between different organisms is directly proportional to their evolutionary relatedness^32^. At the proteome level, comparison was done on the basis of pair-wise conservation of orthologs. Our results indicated that the evolutionary relatedness at both genomic (Fig. 1a) and proteomic levels (Fig. 1b) remained the same. A higher number of conserved proteins in Mbov and Mtb reiterated the close relationship between the two species of Mtb-complex. Our results were also in-line with the 16S rRNA gene sequences based phylogenetic studies on Mbov and Mtb^33^. Also, the number of proteins shared by Mbov and Mtb, with Map (atypical mycobacteria) was less than the proteins shared between Mbov and Mtb. This was similar to the earlier reports based on 16S rRNA based phylogenetic study^34^. During pair-wise comparison of conserved proteins among the four species of *Yersinia*, Ype and Yps shared maximum number of proteins. Similar to Mtb and Mbov, a large number of conserved proteins in Ype and Yps can be attributed to their phylogenetic proximity^3^. An earlier study has also reported that Ype has evolved from Yps^35^, which might be a probable reason behind their closeness. On the other hand Yfr, which is an opportunistic pathogen^36^, shared more proteins with Yen than with Ype or Yps. Earlier phylogenetic studies using multi locus sequence typing have also shown that Ype and Yps belonged to the same cluster and, Yen and Yfr clusters were close to each other^32^. This further confirms their relatedness at the proteome level. We also observed an interesting pattern of shared orthologs of Map with Yen and Yfr. The number of shared orthologs of Map with Yen and Yfr was more, than with Ype and Yps. This might have happened because Map, Yen and Yfr cause gastrointestinal infections and hence occupy the same niche which might have resulted in horizontal transfer of genes among them^37^.

The enrichment of sulfur-containing amino acids and metabolic proteins in intra-genus protein cluster of *Mycobacterium* spp. indicates their importance in survival of *Mycobacterium* spp. Earlier reports also suggested that sulfur containing amino acids help Mtb in sustaining the oxidative stress, nutrient starvation and, in dormancy adaptation^38,39^. Due to presence of sulfur-containing amino acid synthesis pathway proteins exclusively in *Mycobacterium* spp., this pathway was also proposed as a potential target candidate for anti-TB therapy^40^. Analysis of intra-genus protein cluster containing proteins of *Yersinia* spp. revealed conservation of proteins involved in reproduction and mitotic cell cycle. This showed that except for a few, functions of most of the proteins were conserved in both inter- and intra-genus orthologous protein clusters. This also indicates that though sequences of core proteins might have diverged, functionality is retained during evolution. Also, proteins which were unique to a single species and whose orthologs were absent in other species were considered as unique proteins. No significant functional enrichment in species-specific unique proteins was observed because they might belong to different biological pathways, involved in diverse molecular function and reside in different cellular component.

The main objective of this study was to identify proteins that might help in Yps granuloma formation. Therefore, the function of the seven Yps proteins, which were common in Yps and the three *Mycobacterium* spp. were critically analyzed to investigate their probable role in Yps granuloma formation. The protein with the UniProt id A0A0U1QT64 was an uncharacterized protein with an ATPase domain. Since ATPase domains are capable of unfolding the protein substrates, hence proteins harboring ATPase domains are known to be involved in protein degradation. ATPase domains are also essential for intracellular protein degradation because macromolecular assemblies, for *e.g*. proteasome machinery, confine their proteolytic and protease activity in an inner nano-compartment which is accessible only to the unfolded protein substrates^41^. This suggests that proteolytic machinery might be functionally linked to unfolding machinery (AAA – ATPases domain proteins) and are preserved throughout evolution^42,43^. In Mtb, proteostasis network provides protection from different stresses and host immunity. The machinery used for this comprises a complex network of chaperones, proteases, and a eukaryotic-like proteasome (functionally linked AAA – ATPases domain protein family) which helps in evading the host immunity by maintaining the integrity of the mycobacterial proteome^44^. Besides this, AAA – ATPases domain protein family also play a significant role in recognition of ESAT-6 secretion system (ESX-1) secreted virulence factors^45^, which is a type VII secretion system of Mtb and is capable to form pores and rupture phagosomes^46^. This leads to cell toxicity, necrosis and ultimately cell death^47^. On the basis of the functional role of constituent domains in different organism, it can be inferred that this protein might play a probable role in formation of Yps granuloma.

The protein with the UniProt id A0A0U1QTE0 is a functionally uncharacterized protein with a formylglycine-generating sulfatase enzyme domain. Such proteins are reported to be involved in ergotheonine (EGT) synthesis which is a histidine-derived thiol^48^. It reportedly enabled the pathogens in withstanding the host hostile environment during initial phase of infection^49^. EGT-containing proteins are present only in prokaryotes, while plants and animals (including humans) do not produce EGT^50^. Also, macrophages with EGT show an increased cytokine production that enhances Th17 polarization of CD4^+^ T cells. Therefore, it acts as TLR agonist (ligand)^51^ and show immune enhancing property, which causes more cells to come into contact. This indicates that EgtB might be involved in attracting more cells to the site of granuloma formation and thereby help in the process of granuloma formation.

The protein with UniProt id A0A0U1QWK3 was a protein of ABC transporter family (yadG), an integral membrane protein responsible for active transport of ligands across biological membranes^52^. This ABC transporter, ATP–binding protein-encoding gene is present as a pseudogene in Ype (a closely related species of Yps) but is active in Yps^53^. These transporters couple ATP hydrolysis for the uptake and efflux of solutes across the membrane in both bacterial and eukaryotic cells. These are considered as important bacterial virulence factors due to their role in nutrient uptake, secretion of toxins and antimicrobial agents in the host^54^. In *Yersinia* and *Mycobacterium* iron uptake is important for infection and survival in host macrophages^55,56^. Also, ABC transporter system of *Mycobacterium* is similar to the *Yersinia* YbtPQ system^56^. Since, ATP binding cassette transporter proteins are also enriched in tubercular granuloma^30^, it indicates that these proteins might also play an important role in Yps granuloma

The protein with the UniProt id A0A0U1R1R0 is an enzyme, 5-carboxymethyl-2-hydroxymuconate semi aldehyde dehydrogenase (hpaE). The gene encoding this protein is also annotated as a pseudogene in Ype but is actively expressed in Yps^53^. Aldehydes are highly reactive chemical moiety that triggers oxidative stress in both prokaryotes and eukaryotes, that makes them toxic for cells. Enzymes with aldehyde dehydrogenase domain (ALDHs) play an important role in metabolism of both endogenous and exogenous aldehydes. Earlier studies have shown an increased production of ALDHs to cope up with environmental and chemical stress in bacteria^57^. Both *Yersinia* and *Mycobacterium* are intracellular pathogens; hence these bacteria have to combat oxidative stress inside the cell. Previous reports also indicated oxido-reductase enzymes were present in tubercular granuloma^30^. This suggests that this protein might also play an important role in Yps granuloma.

The protein with the UniProt id A0A0U1R1Z2 belongs to the glycosyl hydrolase 10 family of proteins (DJ40_3168). Proteins containing domains of glycoside hydrolase family are present in cellulases (glycoside hydrolases). These proteins play a crucial role in degrading plant cellulose and bacterial cell walls^58^. It has been reported that for transforming from disease causing active state to persistent stage, Mtb dissolves the polysaccharide biofilm in the mammalian host^59^. This also indicates the role of glycoside hydrolases in Mtb virulence. Linkage of Mtb persistence to biofilm also indicates that this protein might also have an important role in Yps granuloma.

The protein with the UniProt id A0A0U1R2S7 is a bacterial regulatory, tetR family protein (yxaF). This gene is also present as pseudogene in Ype but is active in Yps^53^. These proteins contain a TetR DNA-binding, helix-turn-helix (HTH) domain. Proteins containing HTH domains function as DNA-binding transcriptional regulators. These proteins regulate gene expression by binding to the major grove of DNA. These proteins regulate the expression of mycobacterial membrane protein family transporters which are critical for exporting fatty acids and lipidic elements important for mycobacterial virulence^60^. Also, a high rate of lipid transport and metabolism helps in better survival in diverse environments. In Mtb, TetR proteins are found to induce necrosis in lungs^61^. The above-mentioned function of orthologous proteins in Mtb indicates that A0A0U1R2S7 might also play a significant role in necrosis of Yps granuloma.

The protein with the UniProt id A7FMD4 was an enzyme 4-hydroxy-3-methylbut-2-enyl diphosphate reductase (ispH), which is required in DOXP/MEK pathway. The DOXP pathway plays an important role in the pathogenic potential of mycobacterial species. Disruption of DOXP pathway in Mtb hinders its ability to prevent acidification of the phagosome. This results in a decreased potential in intracellular survival^62^. These proteins are present only in pathogenic bacteria, but not in human^63,64^. In *M. avium* subsp. *paratuberculosis, gcpE* mutants were reported to be less efficient in tissue colonization in mice or calves^65,66^, which further confirms the importance of this pathway in virulence. Thus, enzymes of the DOXP pathway have also been proposed as a potential drug target against Mtb^67^.

*Yersinia* and mycobacteria formed two distinct branches on the cladogram drawn on the basis of ANI and PCOP. Despite the differences at genomic and proteomic levels, there are similarities between the granuloma formed by Yps and *Mycobacterium* spp. For example, Yps granuloma is characterized by a central necrosis (caseation) and micro abscess which is also common in tubercular granuloma^24^. Interestingly, functional enrichment revealed that Yps proteins were involved in lipid, phospholipid, isoprenoid, aldehyde and pyruvate metabolism which is similar to the mechanism of granuloma formation in Mtb^30^. Also, lipid metabolism is associated with caseation of granuloma and dissemination of bacteria in the neighboring tissues and organs and, increases the infectivity of bacteria^68^. Despite some similarities, there are also certain subtle differences between the granuloma formed by Yps and *Mycobacterium* spp. For example, the chaperones are well known virulence factors in the formation of tubercular granuloma and are required for bacterial virulence, detoxification and adaptation in Mtb^30,69^. But chaperone proteins were absent in Yps granuloma. Interestingly, protein-protein interaction and metabolic pathway mapping of interacting proteins of the seven common proteins of Yps and *Mycobacterium* spp. revealed that most of these interacting proteins have been proposed as potential drug targets in Mtb (Table 3). Hence, the seven Yps proteins proposed in the present study might be explored as useful drug targets against Yps. Also, an attempt was made to discern if the seven Yps proteins interacted with each other. However, no interaction was observed among these proteins. This might have happened because each of these proteins was involved in a different biological pathway. This also indicates that like Mtb, diverse mechanisms might underlie granuloma formation in Yps^70^.

A comparison of the expression patterns of *in vivo* and *in vitro* derived transcriptome analysis revealed that the Yps early phase infection expression pattern was similar to the *in vitro* expression pattern at 37 °C. Also, the expression pattern of persistent Yps bacteria was approximately similar to that of bacteria grown *in vitro* at 26 °C^26^. Hence, to validate our findings regarding expression of seven proteins we used the RNAseq expression data derived from Yps during *in vitro* growth at 26 °C and 37 °C (GSE55292)^26^. We found that out of the seven proteins, five proteins were also expressed during *in vitro* growth. However, the expression of the remaining two proteins (UniProt ids A0A0U1QT64 and A0A0U1QTE0) could not be ascertained. This might have probably happened because we used Yps strain IP 31758 for our genomic and proteomic analysis, while the transcriptomic data used in our study was based on Yps strain YPIII, which is a plasmid curated strain and, the two proteins, *viz*. UniProt id: A0A0U1QT64 and A0A0U1QTE0 were present on the plasmid. Also, we failed to find the expression in RNAseq data generated by *in vivo* derived total RNA samples. This might be due to a very low abundance of Yps transcripts, which ultimately leads to low coverage of Yps ORFs^26^.

Granuloma formation is primarily a host-defence mechanism which restricts the spread of bacteria. However, some pathogens use it as a protective shell to survive till the advent of favourable conditions. The pathogen resumes its activity and starts multiplication when the conditions become favourable. The best known example of a well-studied pathogen and granuloma is Mtb. Experimental studies in Mtb suggested that RD1 locus proteins^28^, ESAT-6 secretion system proteins^29^ and proteins of intra-macrophage secretome^30^ were mainly involved in Mtb granuloma. Several studies indicated the importance of RD1 locus and ESAT-6 secretion system in Mtb granuloma^71–73^. Mtb strains devoid of RD1 proteins failed to induce Mtb granuloma^74^. Thus, it can be inferred that proteins of the RD1 region, ESAT secretion system and intra-macrophage secretome are important for Mtb granuloma formation. Even after an extensive literature survey we could not find study regarding the mechanistic details of granuloma formation in Yps. Hence, we constructed a composite PPI network of proteins encompassing the proteins of the Mtb RD1 locus, ESAT-6 secretion system and intra-macrophage secretome. The orthologs of the proposed Yps granuloma proteins present in the Mtb proteome were mapped on the composite PPI network map. It was interesting to note that all the Mtb proteins mapped on the composite PPI network showed moderate to strong connections with other proteins of the network. This, suggested that the seven proteins identified in this study might be important for Yps granuloma formation.

To summarize, using a comparative *in silico* proteome analysis of Yps with Map, Mbov and Mtb we identified seven proteins that were absent in Yen, Yfr and Ype. The *in-silico* functional characterization and validation with experimental Mtb granuloma proteins further strengthen our findings that the proposed seven proteins might play some role in Yps granuloma. However, additional experiments involving knocking out of each of these seven proteins are required to confirm their role in Yps granuloma. Additionally, the seven proteins proposed in the present study might not only be the proteins responsible for Yps granuloma and, despite adoption of stringent parameters, many potential granuloma proteins might have been missed. We understand that a detailed functional characterization of Yps proteins is required to unravel the complex mechanisms underlying Yps granuloma formation. Nevertheless, our study provides some useful insights and can serve as a basis for further studies on Yps granuloma.

## Materials and Methods

### Genomes and proteomes used for analysis

The proteome and genome data sets used in the present study, were obtained from UniProtKB (release 2017_09)^75^ (Table 1) and NCBI (http://www.ncbi.nlm.nih.gov) respectively. The accession numbers of proteomes and genomes of the seven microbes used in the present work are as follows: Mtb (UniProt ID: UP000001584; NCBI ID: NC_000962.3), Mbov (UniProt ID: UP000001419; NCBI ID: AP010918.1), Map (UniProt ID: UP000000580; NCBI ID: NC_002944.2), Ype (UniProt ID: UP000000815; NCBI ID: NC_003143.1), Yen (UniProt ID: UP000000642; NCBI ID: AM286415.1), Yps (UniProt ID:UP000002412; NCBI ID: NZ_CP008943.1), and Yfr (UniProt ID: UP000005500; NCBI ID: NZ_CP009364.1).

### Determination of relatedness and distinctiveness among different species

To determine the genomic relatedness among the seven bacterial species, Average Nucleotide Identity (ANI) was calculated using OrthoANI^76^. To estimate the evolutionary distance among proteomes of different species, the percentage of conserved proteins (PCOP) was calculated^77^. The values of ANI and PCOP were used to construct the Neighbor-Joining (NJ) tree^78^ using MEGA^79^.

### Identification of orthologous proteins

All the possible combinations of the seven proteomes were made and, orthologous proteins in each group were identified. To find orthologous proteins we used InParanoid (version 4.1) at default parameters. InParanoid performs reciprocal BLAST and labels protein sequences based on sequence similarity as orthologs^80^. For each ortholog, InParanoid provides bit score in the range of 0.5–1. In this study we considered two proteins as orthologs, if the InParanoid score was ≥0.8.

### Clustering of orthologous sequences

On the basis of the number of proteomes in which a set of orthologous proteins was present, orthologous sequences were categorized into mutually exclusive clusters. Proteins of each cluster represented a specific chunk of proteins that was not shared by other clusters. For example proteins in inter-genus ortholog cluster contained proteins that were present in all the seven proteomes. Similarly, intra-genus ortholog cluster contained proteins, which were present only in *Yersinia* or *Mycobacterium*.

### Functional enrichment of proteins

Functional annotation of each protein cluster was done by assigning them gene ontology (GO) terms. The GO terms were retrieved from the Gene Ontology Consortium^81^. The functional enrichment of orthologous protein clusters was done using topGO tools (v2.24.0) of Bioconductor package^82^. In the present work, high-level view of GO terms, namely GO-slim terms, was used to determine the enriched functions. These terms were extracted from the GO annotation dataset by GO Slim Mapper OWL Tool (https://github.com/owlcollab/owltools.git). During enrichment, all the proteins present in the seven proteomes were divided into seven broad categories (or “test datasets”) and a unique background was used during enrichment of each test-dataset. The enrichment was done on cluster of orthologous sequences (or “test datasets”) and a unique background was used during enrichment of each test-dataset.

#### Set I (inter-genus conserved proteins)

Inter genus conserved set included proteins that were conserved across all the seven proteomes. During functional enrichment of this category of proteins, combined GO-slim terms of all seven complete proteomes was used as background.

#### Set II (intra-genus conserved proteins)

Intra genus conserved set included the proteins present in all species of genus *Mycobacterium* or *Yersinia*. During functional enrichment of this group of proteins, collective GO-slim terms of proteome of respective genus was used as background.

#### Set III (Conserved in Ype and *Mycobacterium* spp.)

This contains the proteins which were common in Ype and the three *Mycobacterium spp*., The functional enrichment of proteins of Set III proteins were determined against using all four *Yersinia* spp. as background.

#### Set IV (Conserved in Yen and *Mycobacterium* spp.)

The functional enrichment of proteins of Yen whose orthologs were present in all *Mycobacterium* spp. (as test-dataset) were determined against all the four *Yersinia* spp. (as background).

#### Set V (Yfr with *Mycobacterium spp*.)

enriched function of Yfr proteins, whose orthologs were present in *Mycobacterium* spp. (as test-dataset) were determined against all the four *Yersinia* spp. (as background).

#### Set VI (Yps with *Mycobacterium* spp.)

enriched function of Yps proteins which showed orthology with the proteins of *Mycobacterium* spp. (as test-dataset) were determined against all the four *Yersinia* spp. (as background).

#### Set VII (with-in the species)

This set includes proteins unique to a particular species. To find functional enrichment in these proteins, GO-slim terms retrieved from the complete proteome of the same species were used as the background.

### Characterization of Yps proteins involved in granuloma formation

Since, the aim of the current study was identification of Yps proteins involved in granuloma formation, hence only those proteins of Yps whose orthologs were present in the MTB- complex members, were functionally characterized. For functional annotation a three-step process was followed: (a) domain information of each protein was collected from UniProtKB; (b) the protein and their interaction partners were identified using STRING database (https://string-db.org/)^83^ and characterized; (c) the metabolic pathways in which the interacting protein partners were involved were identified using the KEGG database^84^ and, (d) information on whether the interacting proteins and/or pathways have been used as drug targets was retrieved from the published literature.

### Mapping of Yps predicted proteins on composite PPI interaction network of experimentally identified Mtb granuloma proteins

A composite interaction network of RD1 locus proteins^28^, ESAT-6 secretion system proteins^29^ and intra-macrophagic secretome of Mtb^30^ was created using STRING database, at confidence score 0.150. The Mtb orthologs of the proposed seven Yps granuloma proteins were mapped on this interaction network.
